# Supplementary figures and images for: Spatial transcriptomics elucidates localized immune responses in atherosclerotic coronary artery
Source: EMBO Mol Med. 2025 Aug 22;17(10):2827–46. doi: 10.1038/s44321-025-00280-w (PMC12514278; doi:10.1038/s44321-025-00280-w)

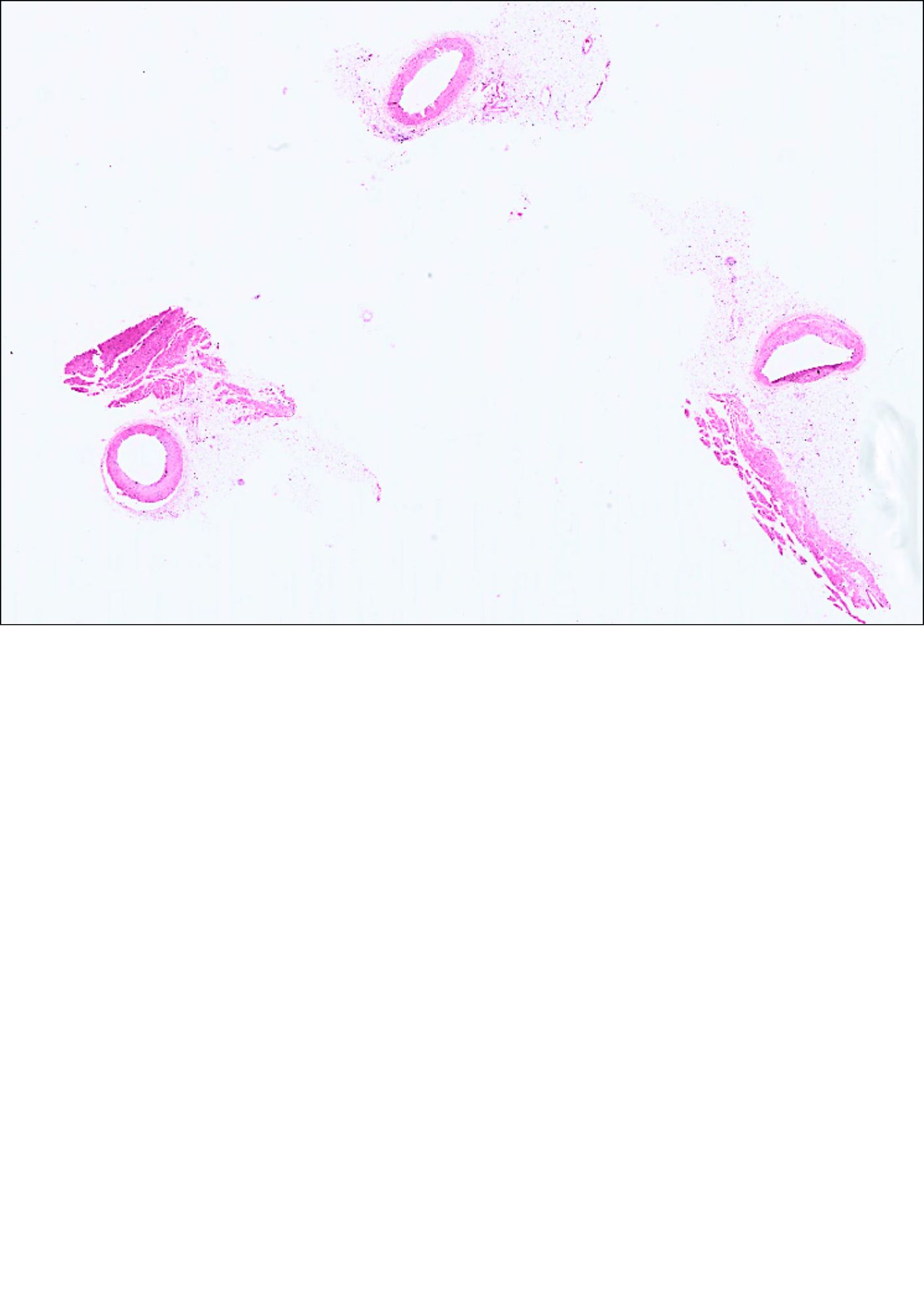

Supplement: Supplementary file 3 — Source data Fig. 1 [file 44321_2025_280_MOESM3_ESM.zip › Figure 1A_mild.jpg]

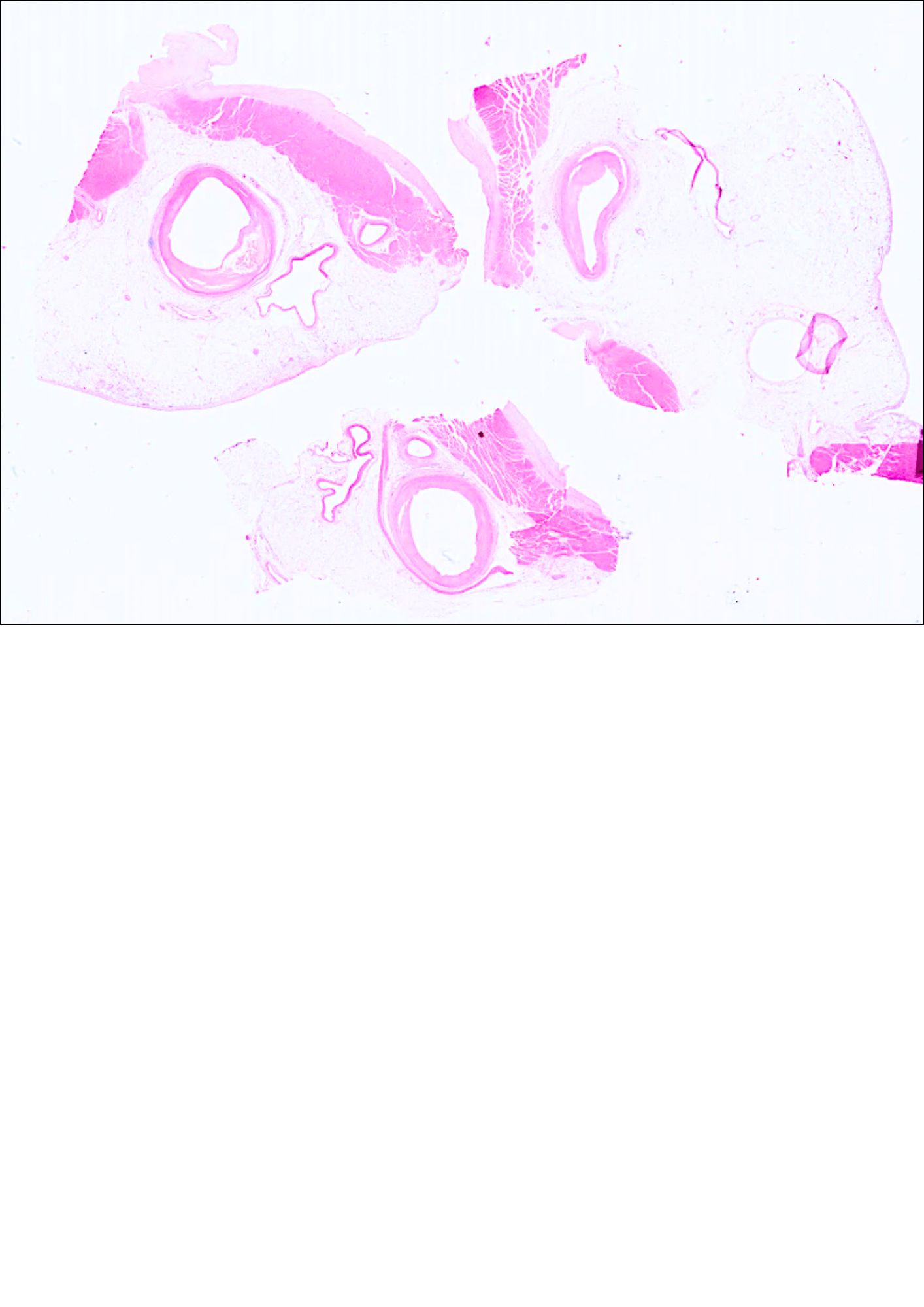

Supplement: Supplementary file 3 — Source data Fig. 1 [file 44321_2025_280_MOESM3_ESM.zip › Figure 1A_moderate.jpg]

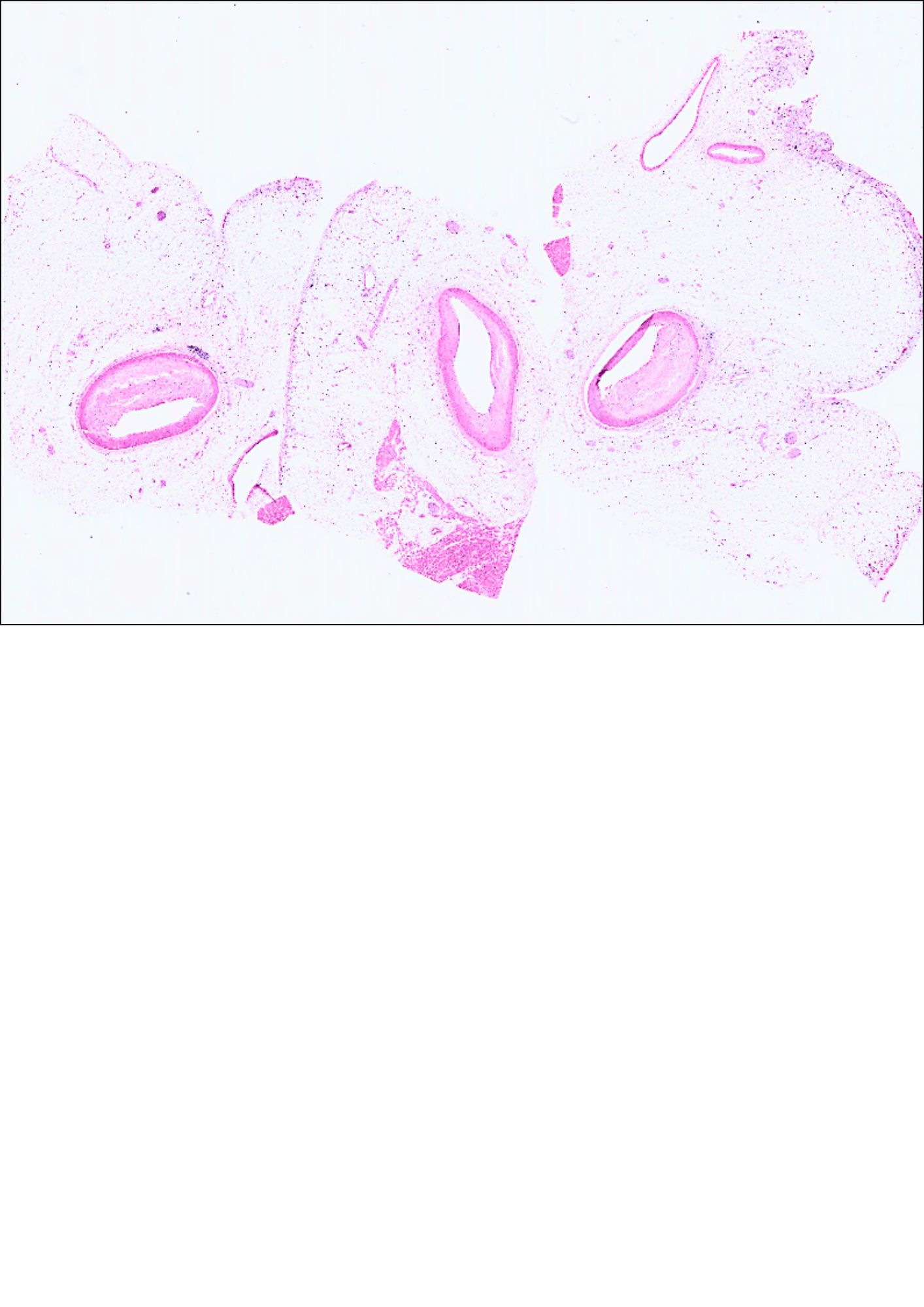

Supplement: Supplementary file 3 — Source data Fig. 1 [file 44321_2025_280_MOESM3_ESM.zip › Figure 1A_severe.jpg]

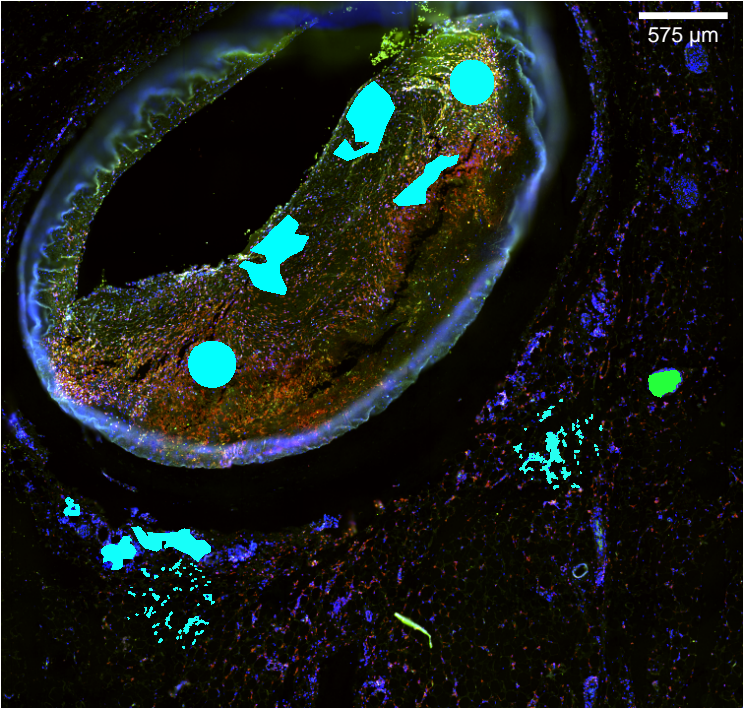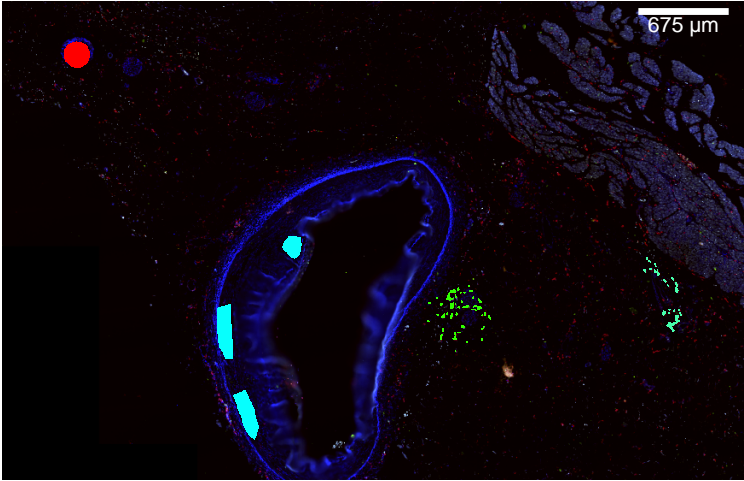

Supplement: Supplementary file 4 — Source data Fig. 2 [file 44321_2025_280_MOESM4_ESM.zip › Figure 2E_ APOD gene expression in severe and mild samples.pdf]

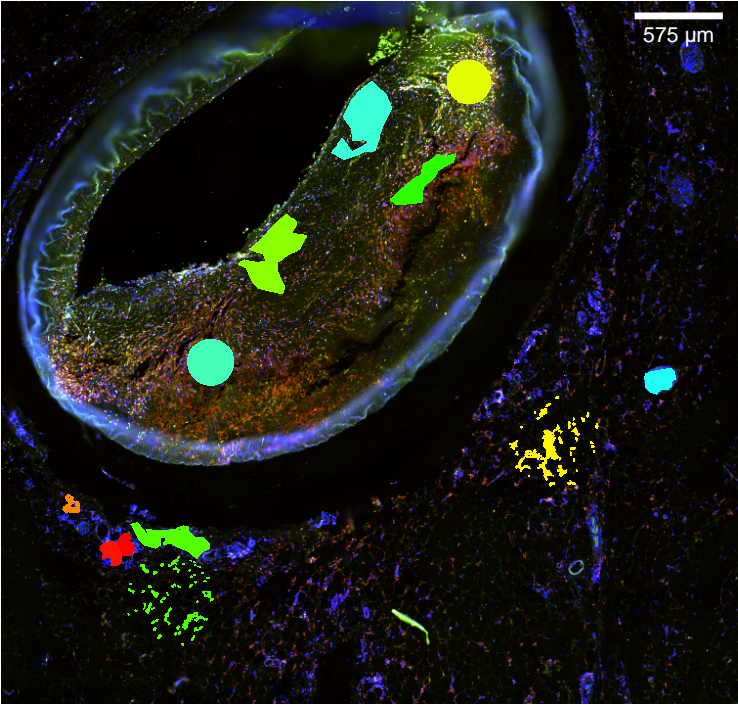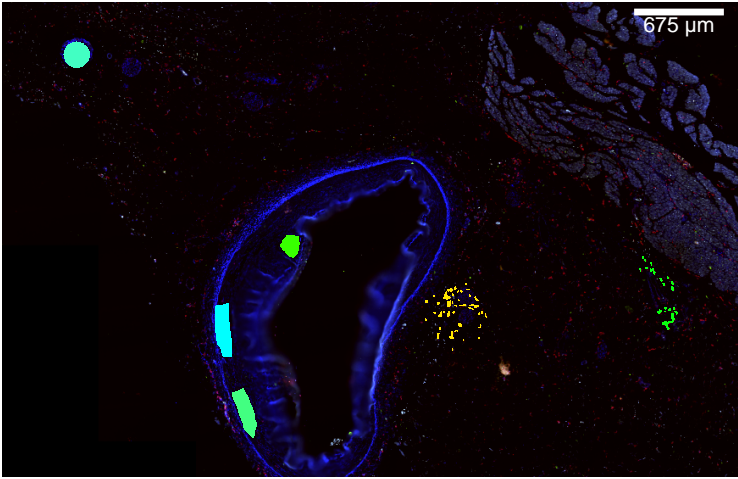

Supplement: Supplementary file 4 — Source data Fig. 2 [file 44321_2025_280_MOESM4_ESM.zip › Figure 2E_SPOCK2 gene expression in severe and mild samples.pdf]

Cell coordinates in XY space

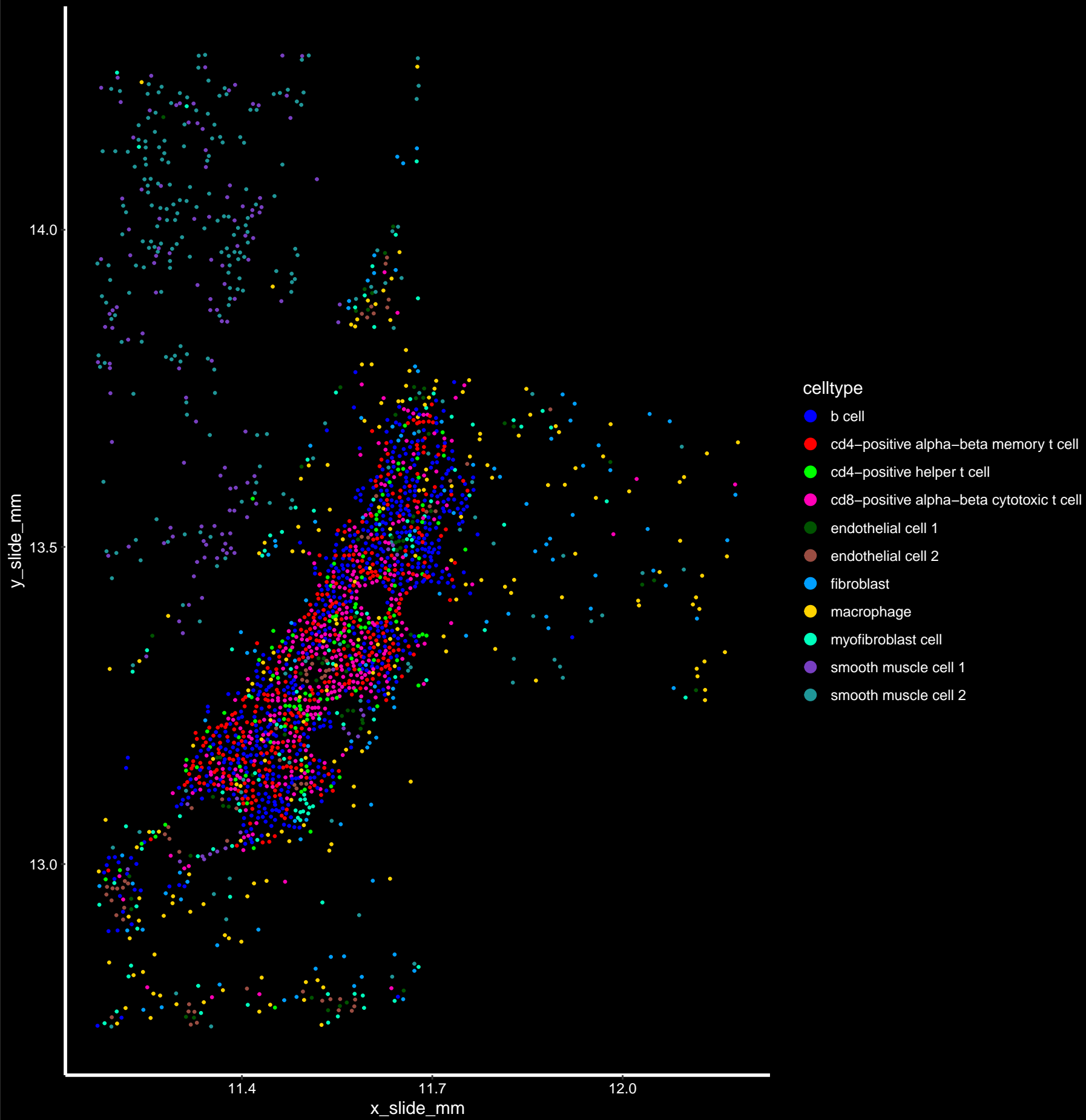

Supplement: Supplementary file 6 — Source data Fig. 5 [file 44321_2025_280_MOESM6_ESM.zip › Figure5E_ELS.pdf]

Cell coordinates in XY space

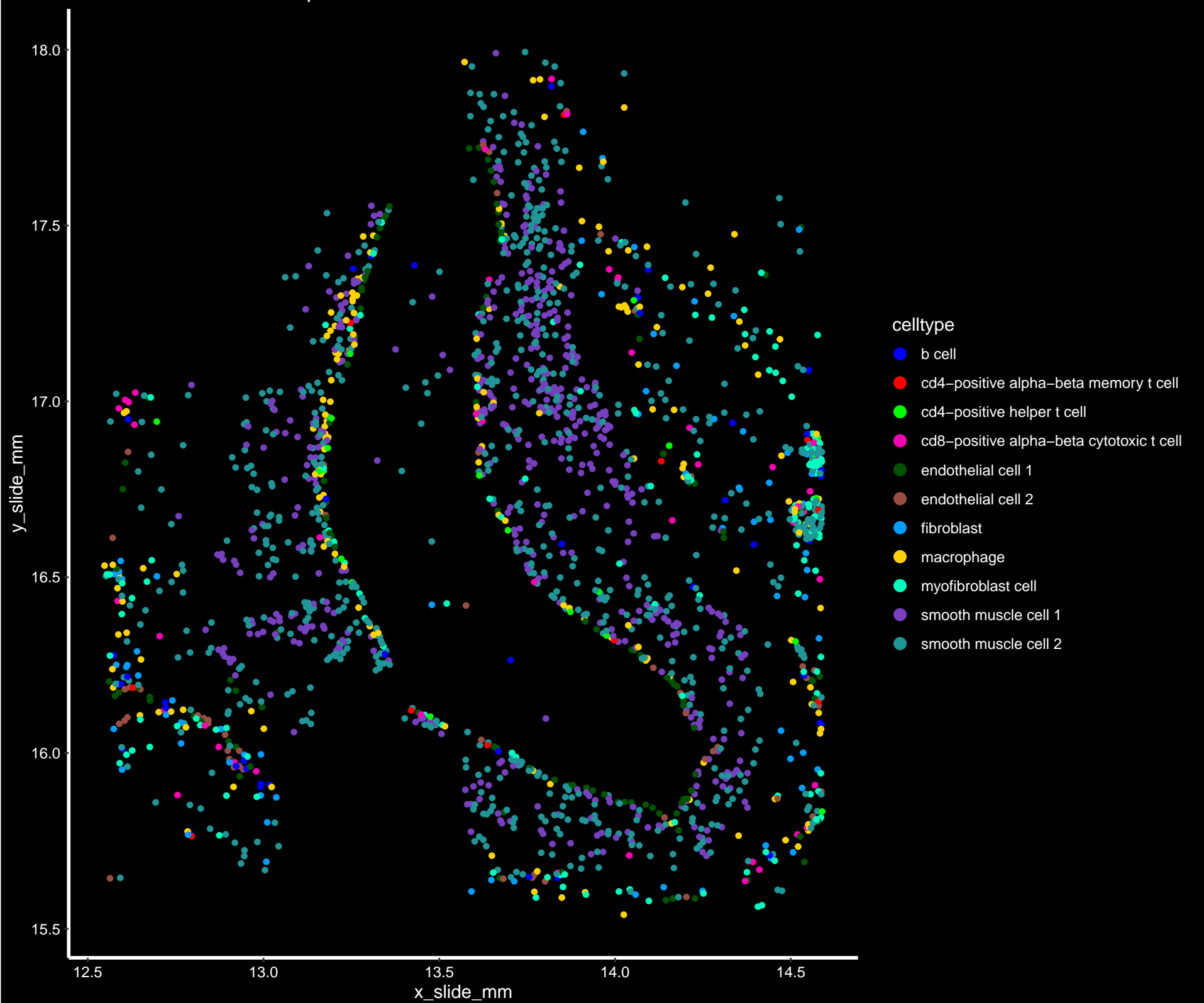

Supplement: Supplementary file 6 — Source data Fig. 5 [file 44321_2025_280_MOESM6_ESM.zip › Figure5E_Mild.pdf]

Cell coordinates in XY space

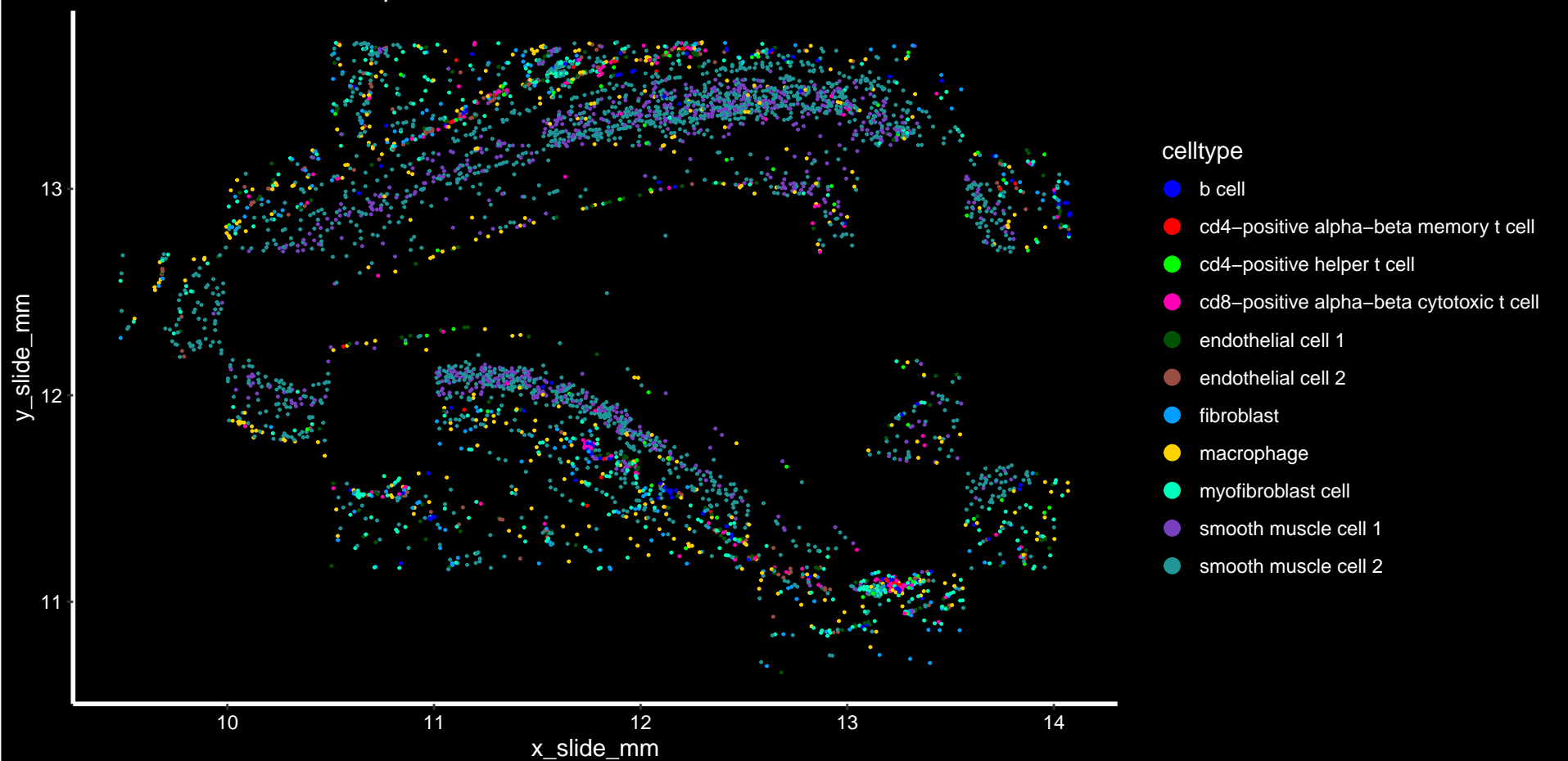

Supplement: Supplementary file 6 — Source data Fig. 5 [file 44321_2025_280_MOESM6_ESM.zip › Figure5E_Moderate.pdf]

Cell coordinates in XY space

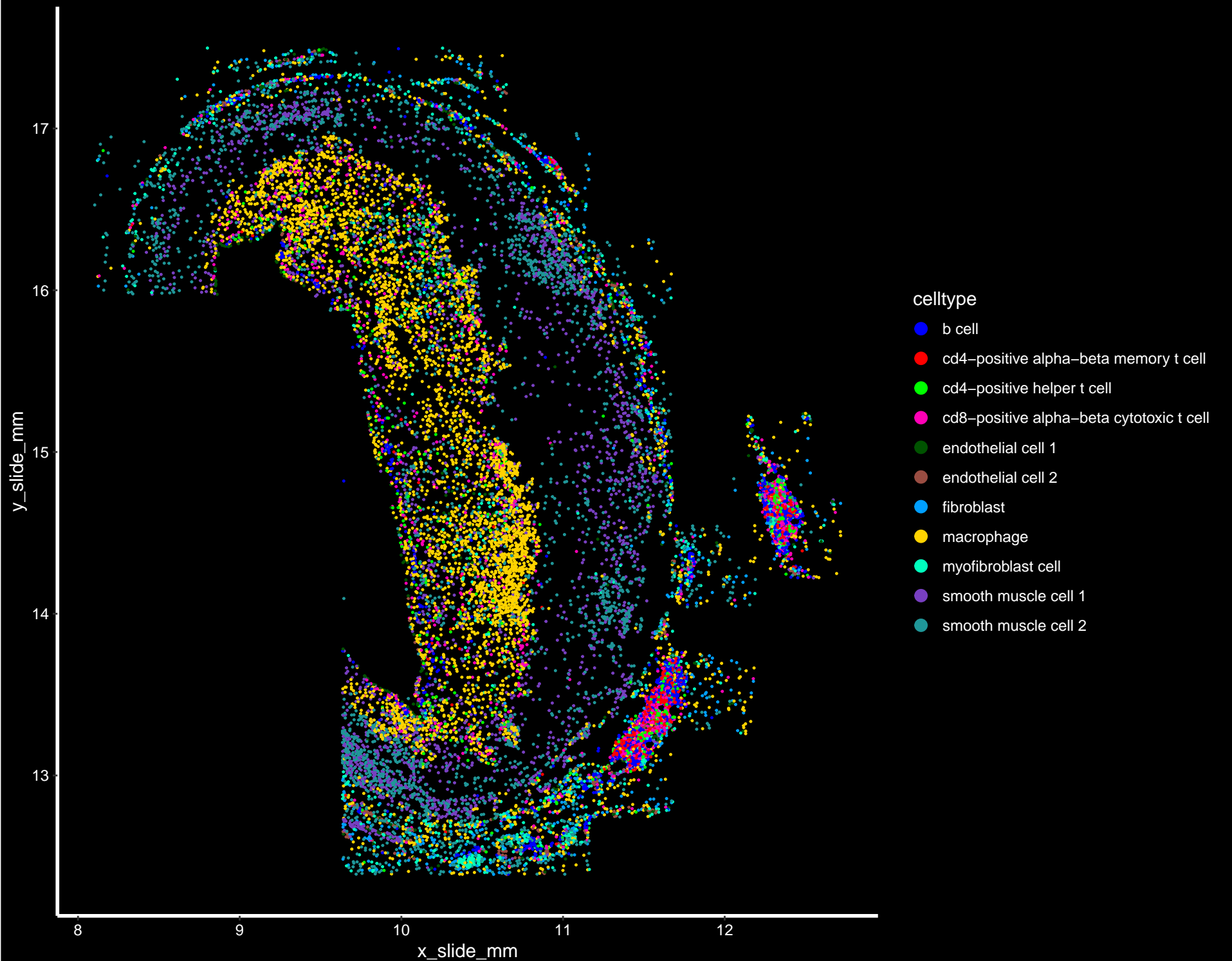

Supplement: Supplementary file 6 — Source data Fig. 5 [file 44321_2025_280_MOESM6_ESM.zip › Figure5E_Severe.pdf]

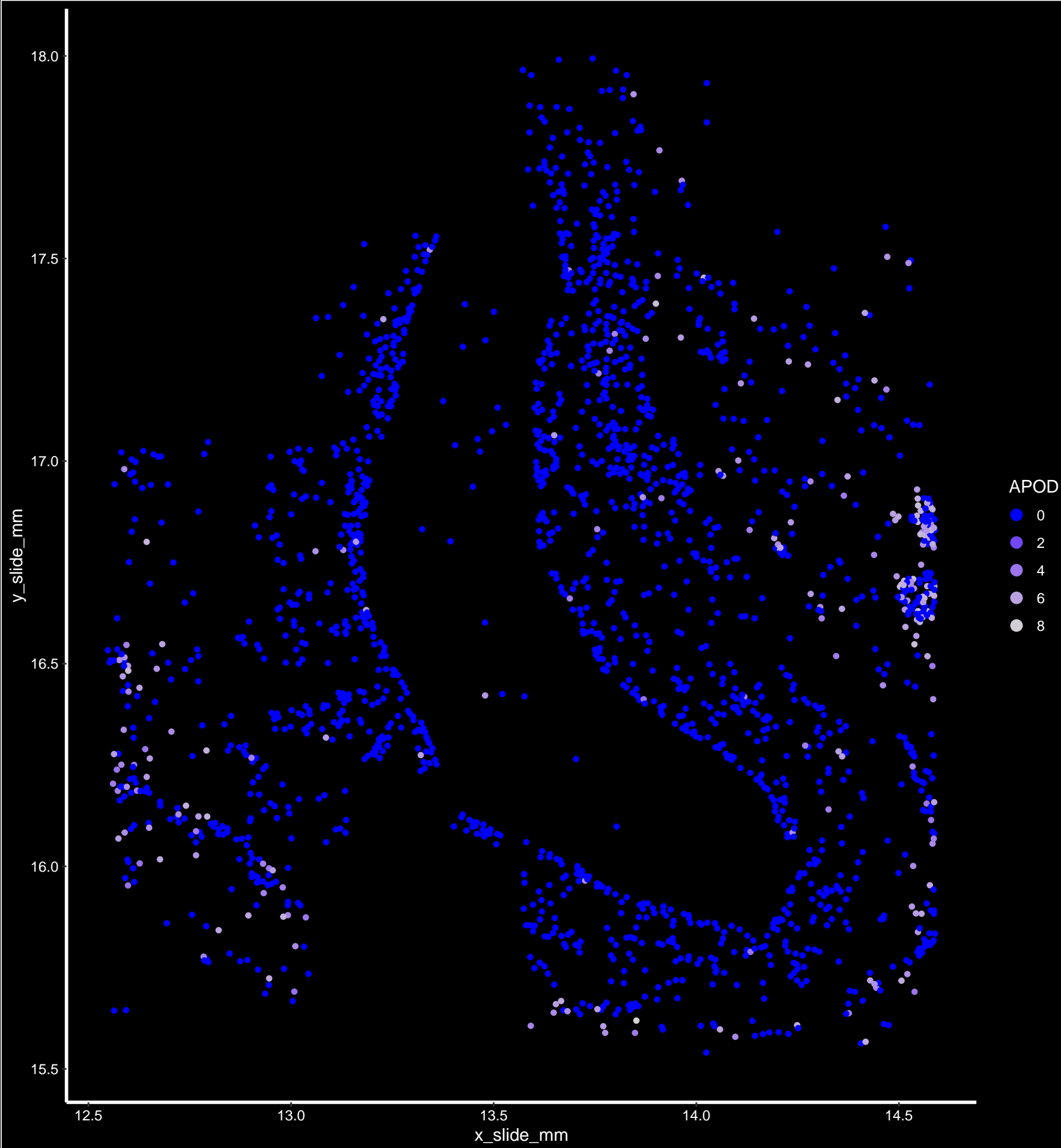

Supplement: Supplementary file 6 — Source data Fig. 5 [file 44321_2025_280_MOESM6_ESM.zip › Figure5F_Mild-APOD.pdf]

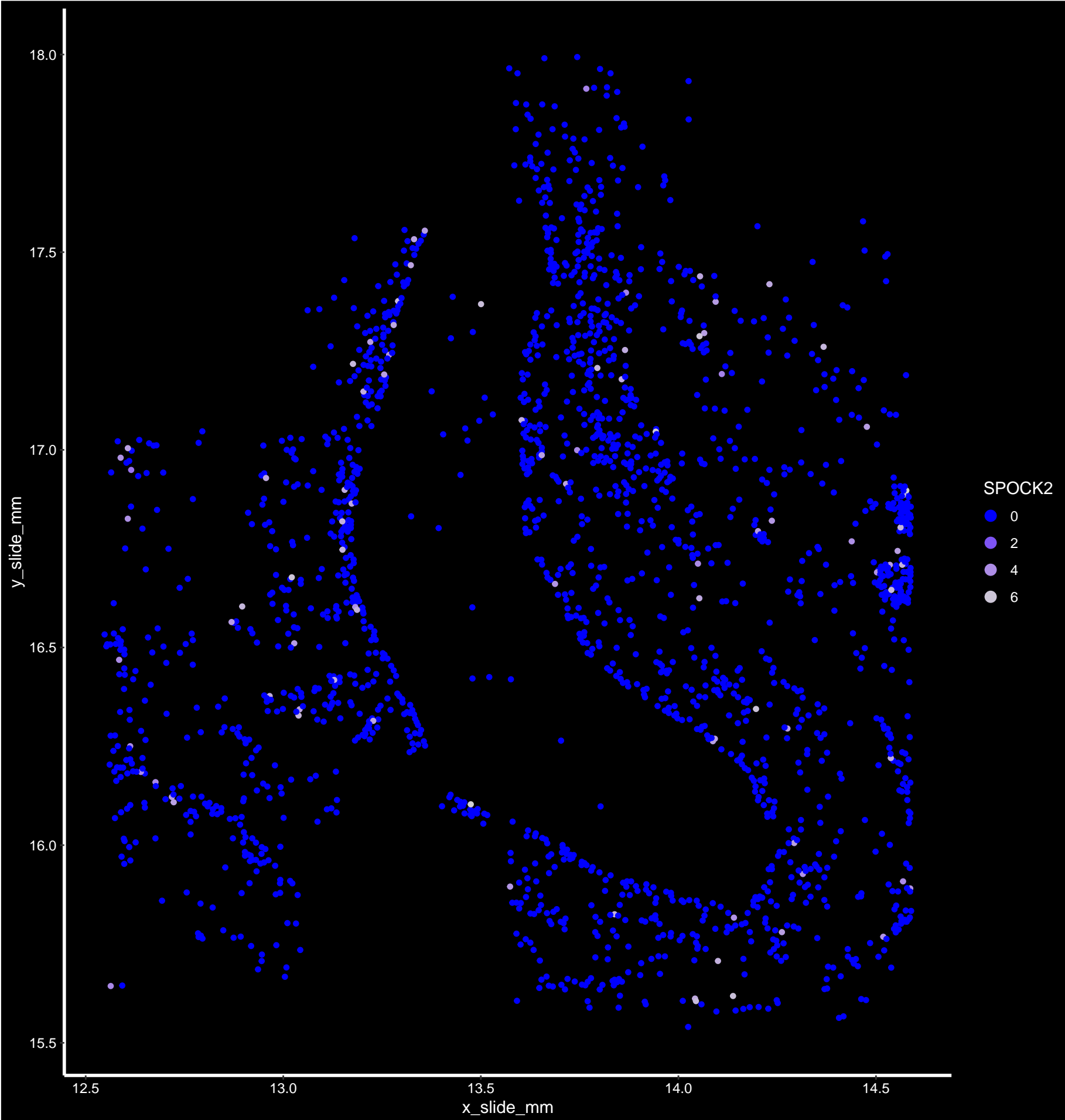

Supplement: Supplementary file 6 — Source data Fig. 5 [file 44321_2025_280_MOESM6_ESM.zip › Figure5F_Mild-SPOCK2.pdf]

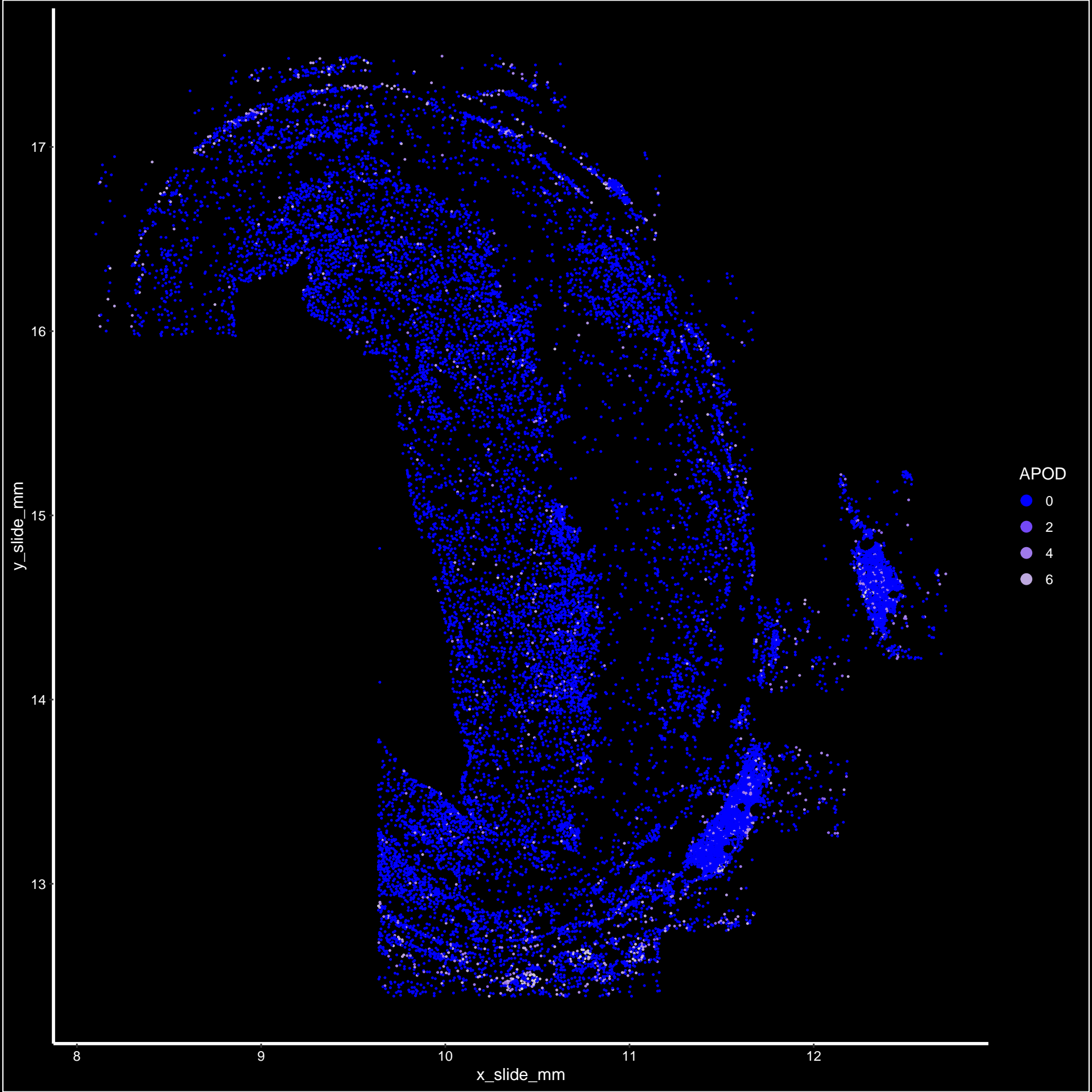

Supplement: Supplementary file 6 — Source data Fig. 5 [file 44321_2025_280_MOESM6_ESM.zip › Figure5F_Severe-APOD.pdf]

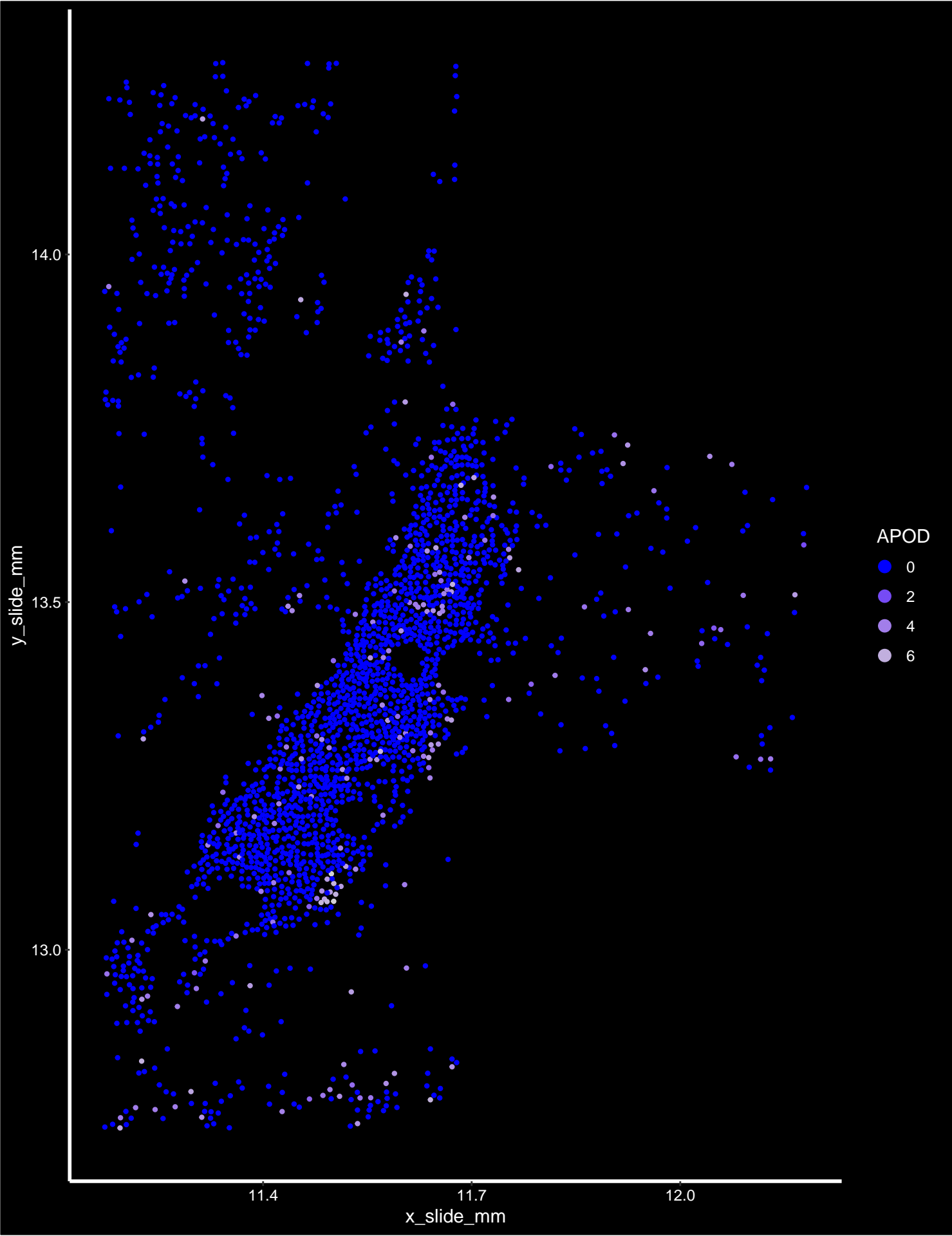

Supplement: Supplementary file 6 — Source data Fig. 5 [file 44321_2025_280_MOESM6_ESM.zip › Figure5F_Severe-ELS-APOD.pdf]

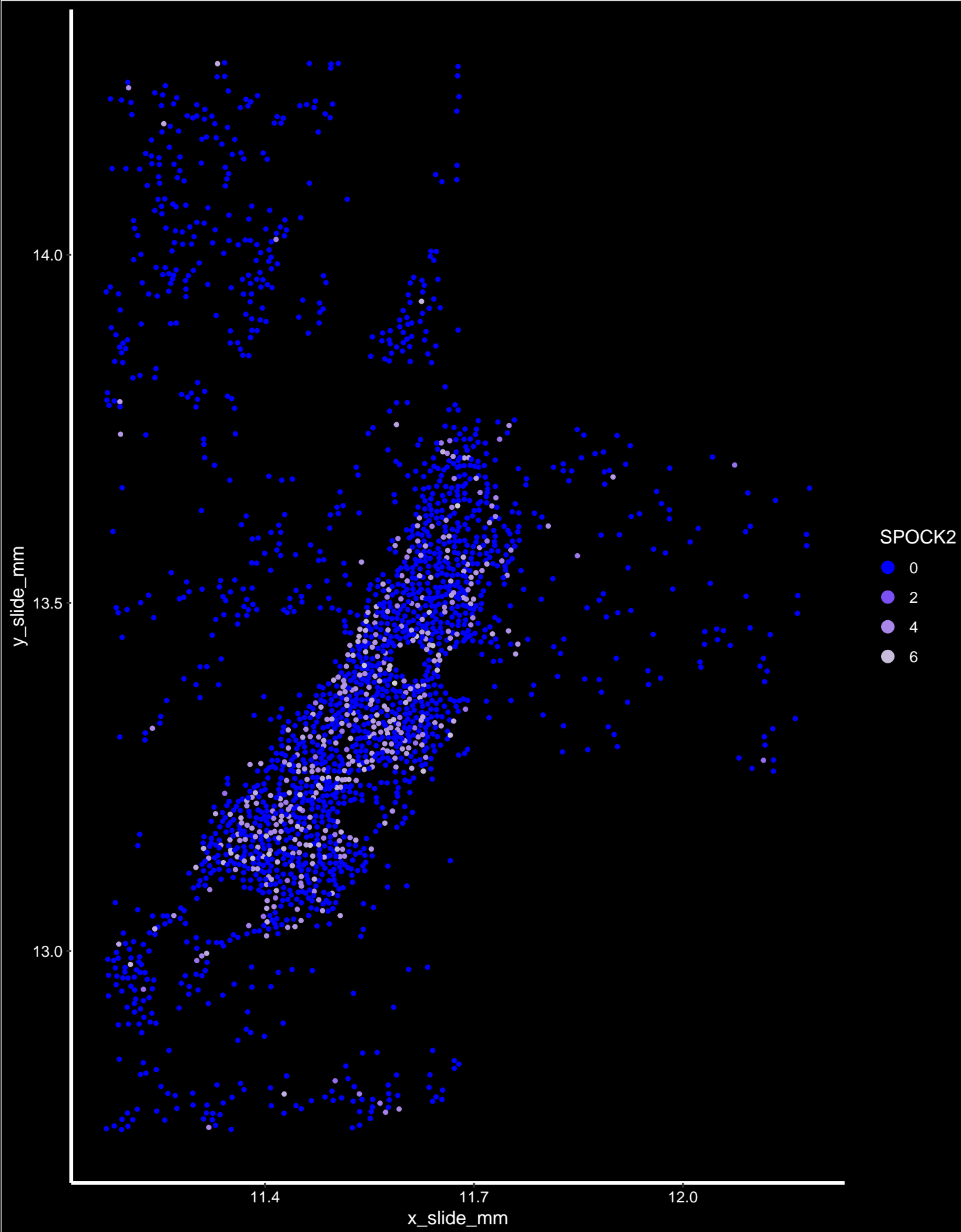

Supplement: Supplementary file 6 — Source data Fig. 5 [file 44321_2025_280_MOESM6_ESM.zip › Figure5F_Severe-ELS-SPOCK2.pdf]

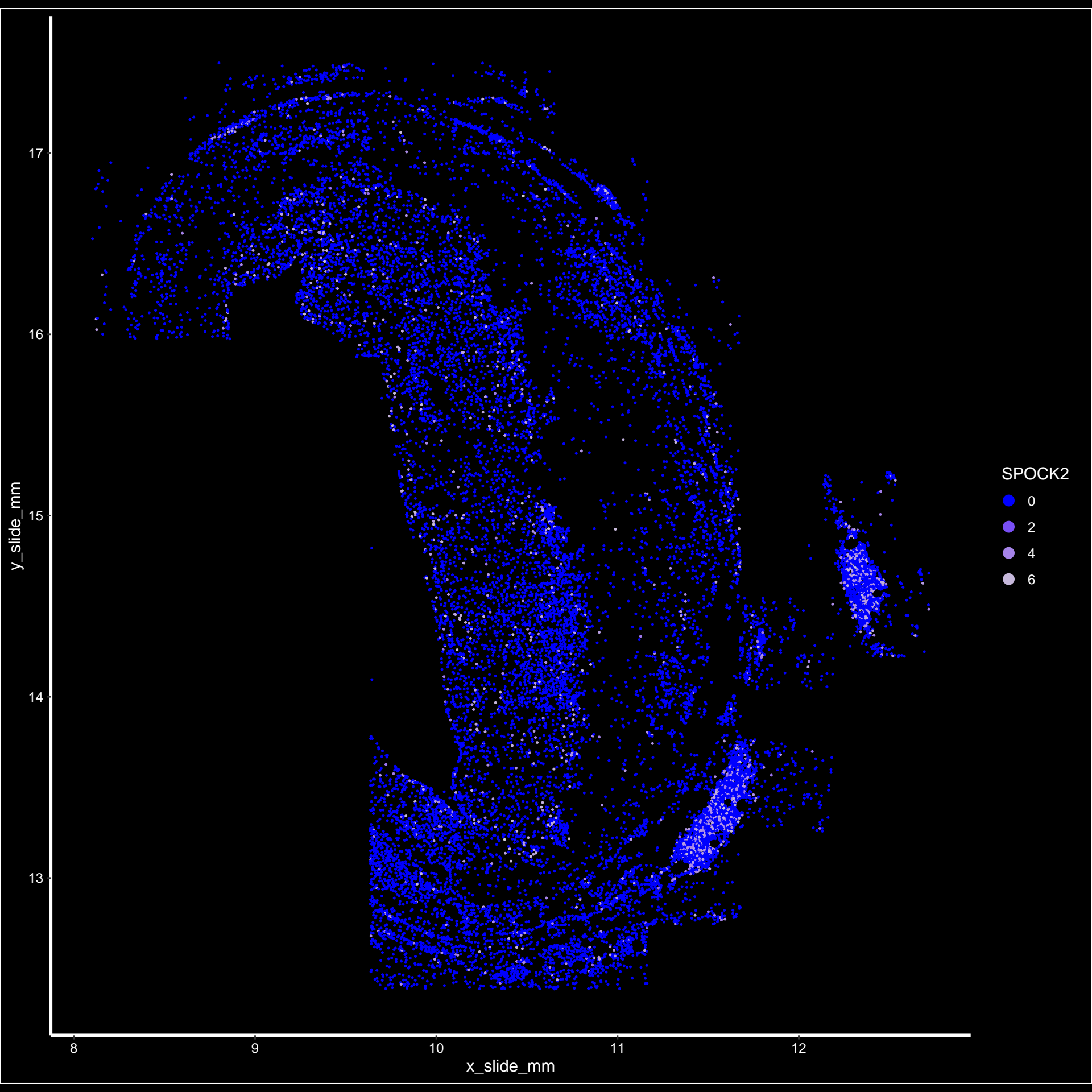

Supplement: Supplementary file 6 — Source data Fig. 5 [file 44321_2025_280_MOESM6_ESM.zip › Figure5F_Severe-SPOCK2.pdf]

650  $\mu$ m

ANXA2

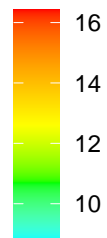

Supplement: Supplementary file 8 — Source data Fig. 7 [file 44321_2025_280_MOESM8_ESM.zip › Figure7A_ANXA2 expression in severe artery.pdf]

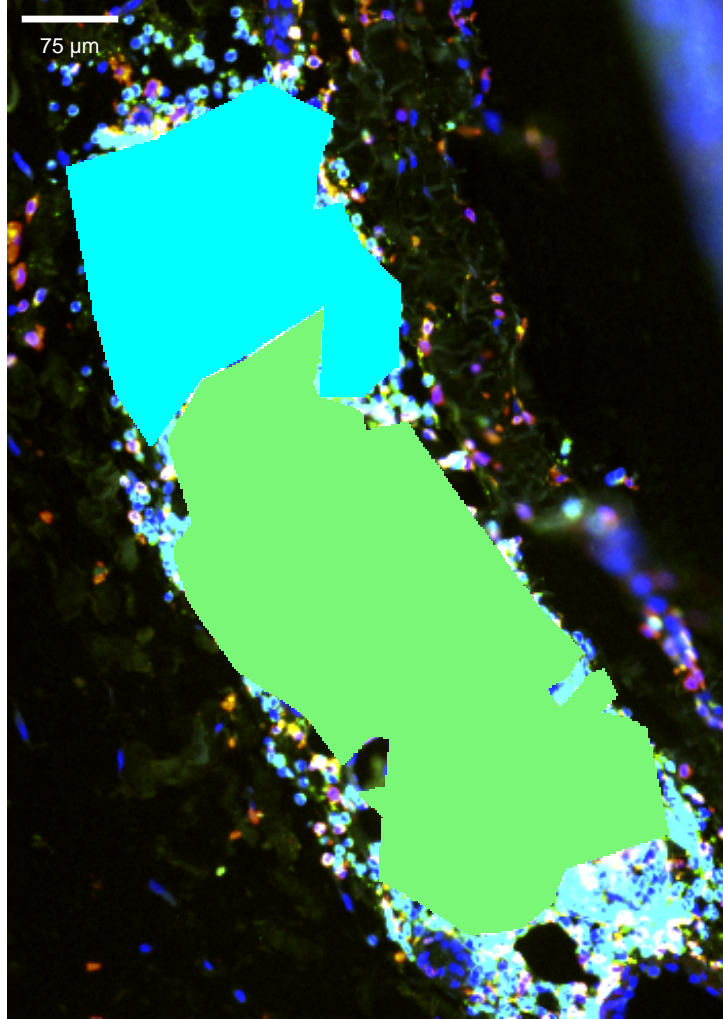

ANXA2

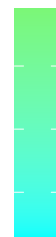

9.75

9.50

9.25

Supplement: Supplementary file 8 — Source data Fig. 7 [file 44321_2025_280_MOESM8_ESM.zip › Figure7A_ANXA2 expression in TLS.pdf]

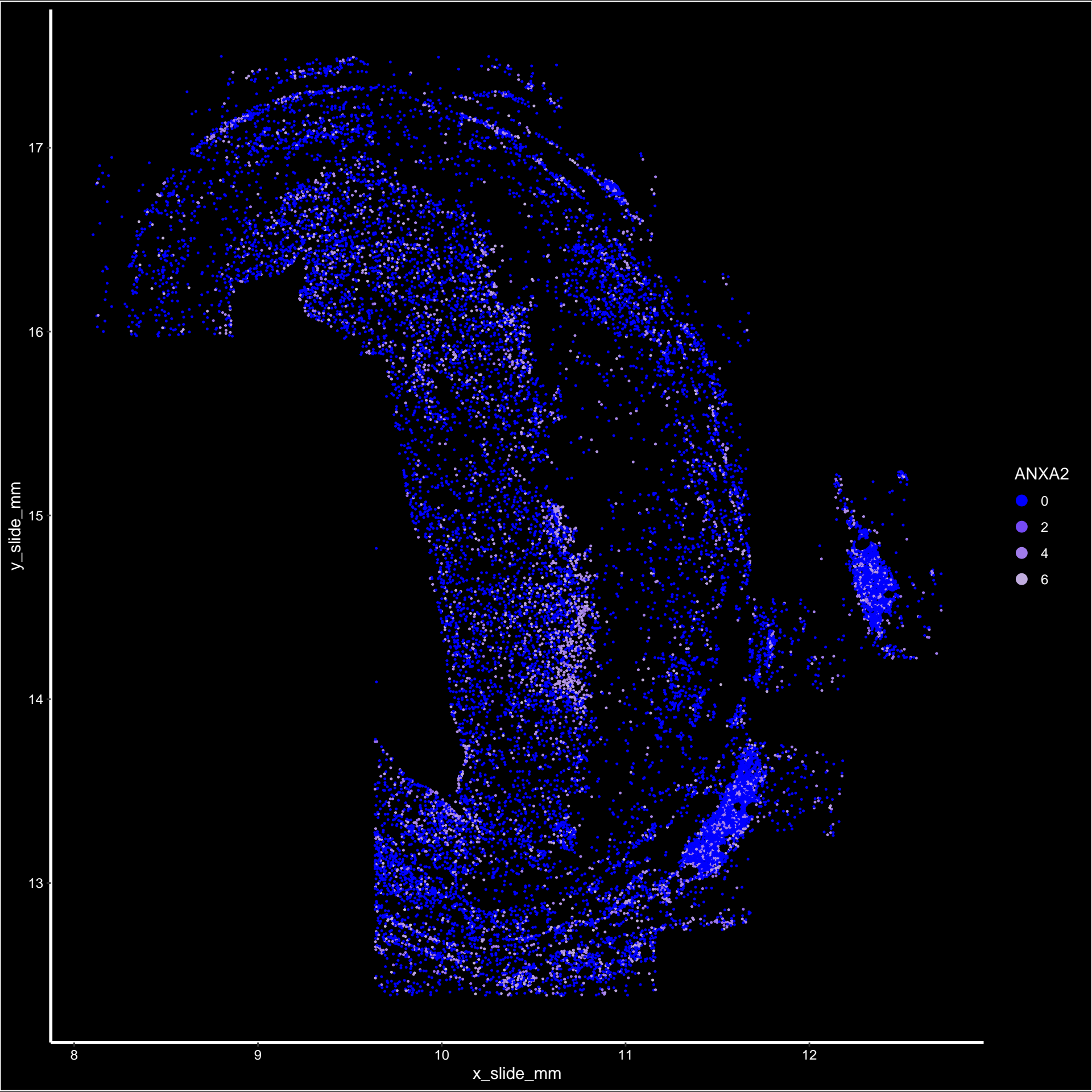

Supplement: Supplementary file 8 — Source data Fig. 7 [file 44321_2025_280_MOESM8_ESM.zip › Figure7A_S496626-ANXA2.pdf]

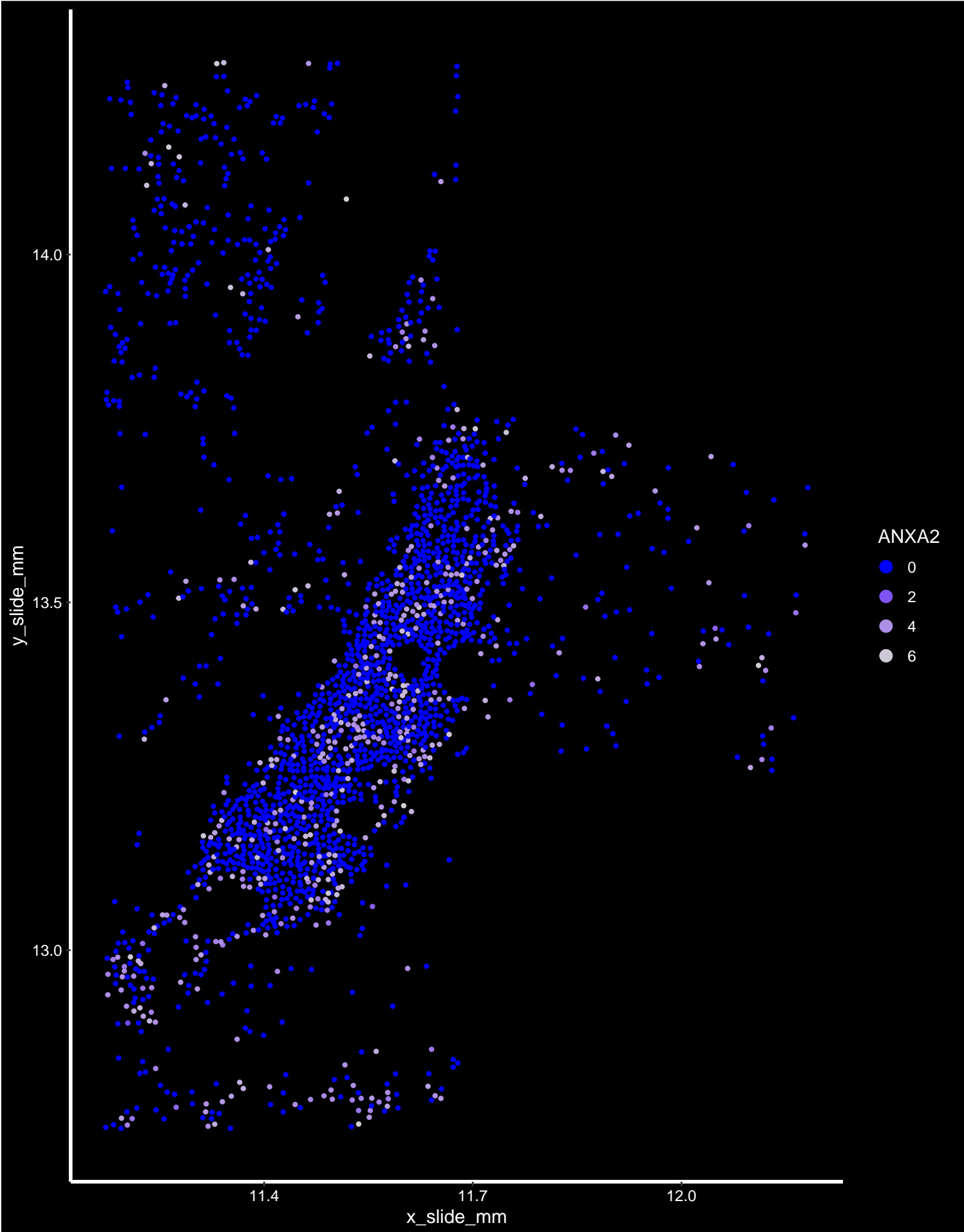

Supplement: Supplementary file 8 — Source data Fig. 7 [file 44321_2025_280_MOESM8_ESM.zip › Figure7A_S496626-ELS-ANXA2.pdf]
