# Supplementary material for: Spatial transcriptomics elucidates localized immune responses in atherosclerotic coronary artery
Source: EMBO Mol Med. 2025 Aug 22;17(10):2827–46. doi: 10.1038/s44321-025-00280-w (PMC12514278; doi:10.1038/s44321-025-00280-w)
Supplement: Supplementary file 9 — Expanded View Figures [file 44321_2025_280_MOESM9_ESM.pdf]

Expanded View Figures

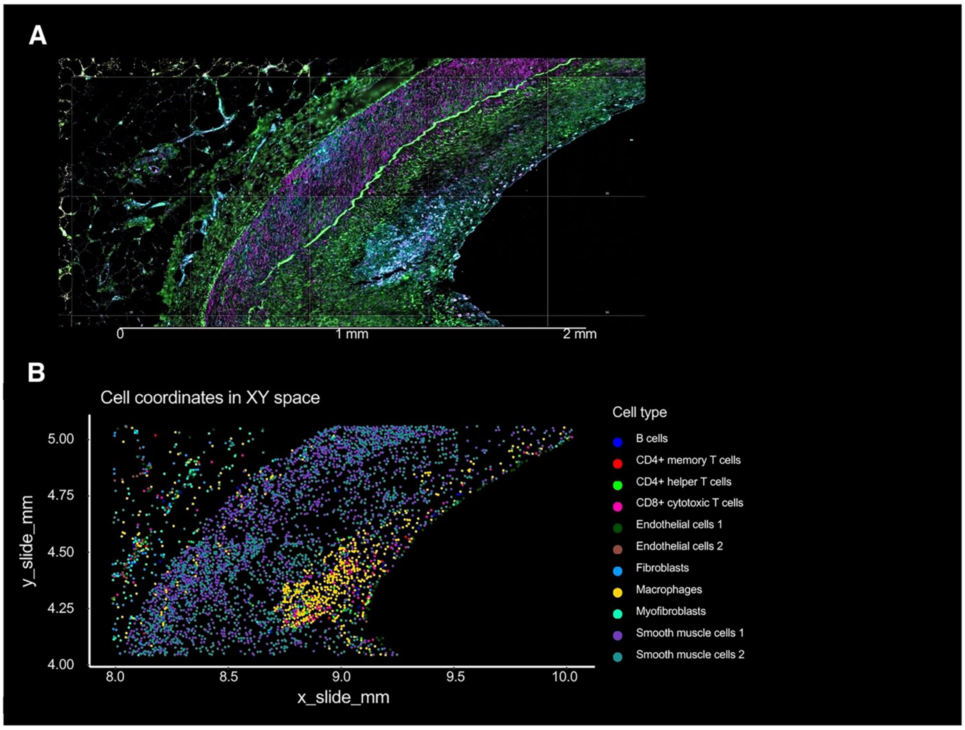

Figure EV1. Protein immunofluorescence staining and RNA-driven cell typing of the same region within an atherosclerotic vessel.

(**A**) Immunofluorescence scan generated by CosMx™ showing the full thickness of the coronary artery and (**B**) respective cell typing equivalent following supervised cell typing using Census vasculature signature.

 © The Author(s)

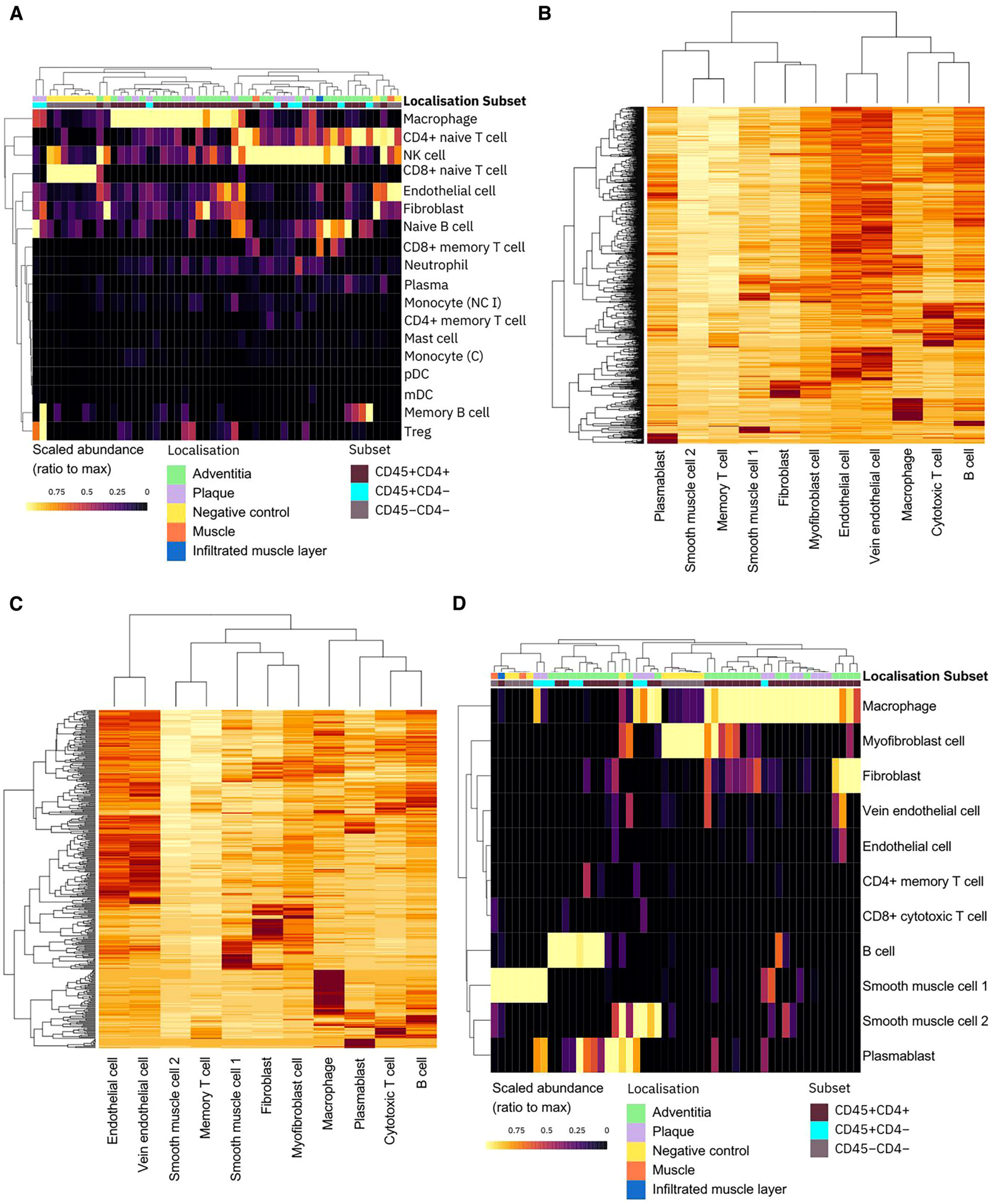
A
Localisation Subset
Macrophage
CD4+ naive T cell
NK cell
CD8+ naive T cell
Endothelial cell
Fibroblast
Naive B cell
CD8+ memory T cell
Neutrophil
Plasma
Monocyte (NC I)
CD4+ memory T cell
Mast cell
Monocyte (C)
pDC
mDC
Memory B cell
Treg
Scaled abundance (ratio to max)
0.75 0.5 0.25 0
Localisation
Adventitia
Plaque
Negative control
Muscle
Infiltrated muscle layer
Subset
CD45+CD4+
CD45+CD4−
CD45−CD4−
B
Plasmablast
Smooth muscle cell 2
Memory T cell
Smooth muscle cell 1
Fibroblast
Myofibroblast cell
Endothelial cell
Vein endothelial cell
Macrophage
Cytotoxic T cell
B cell
C
Endothelial cell
Vein endothelial cell
Smooth muscle cell 2
Memory T cell
Smooth muscle cell 1
Fibroblast
Myofibroblast cell
Macrophage
Plasmablast
Cytotoxic T cell
B cell
D
Localisation Subset
Macrophage
Myofibroblast cell
Fibroblast
Vein endothelial cell
Endothelial cell
CD4+ memory T cell
CD8+ cytotoxic T cell
B cell
Smooth muscle cell 1
Smooth muscle cell 2
Plasmablast
Scaled abundance (ratio to max)
0.75 0.5 0.25 0
Localisation
Adventitia
Plaque
Negative control
Muscle
Infiltrated muscle layer
Subset
CD45+CD4+
CD45+CD4−
CD45−CD4−

◀ **Figure EV2. A comparison of spatial deconvolution estimates using inbuilt matrices versus CosMx™ derived signatures.**

(**A**) Heatmap of spatial deconvolution estimates using the inbuilt GeoMx® reference matrix 'safeTME'. Scaled abundances are shown as a ratio to the maximum value are displayed across five tissue localisations (adventitia, plaque, negative control, muscle and infiltrated muscle layer). 'Subsets' correspond to ROIs segmented on the GeoMx® platform and are highlighted. Cell types present in the safeTME matrix are indicated as rows. (**B**) Heatmap of the CosMx™-derived cell signature matrix. Census-annotated cell populations from the CosMx™ are represented as columns with rows representing genes on the CosMx™ platform. Genes are scaled from red to white, with red indicating a higher expression. (**C**) Heatmap of the genes present in the GeoMx® dataset from the CosMx™-derived cell signature matrix. (**D**) Heatmap of spatial deconvolution estimates using the CosMx™-derived matrix. Cell types present in the CosMx™-matrix are indicated as rows.

 © The Author(s)
